# Hydrogenation of High Molecular Weight Bisphenol A Type Epoxy Resin BE503 in a Functional and Greener Solvent Mixture Using a Rh Catalyst Supported on Carbon Black

**DOI:** 10.3390/polym12112513

**Published:** 2020-10-28

**Authors:** Bo-Xin Lai, Saurav Bhattacharjee, Yi-Hao Huang, An-Bang Duh, Ping-Chieh Wang, Chung-Sung Tan

**Affiliations:** 1 Department of Chemical Engineering, National Tsing Hua University, No. 101, Section 2, Guangfu Road, East District, Hsinchu City 30013, Taiwan; paul84120@gmail.com (B.-X.L.); s105032892@m105-mail.nthu.edu.tw (S.B.); andy85519@gmail.com (Y.-H.H.); 2 Chang Chun Plastics Co., Ltd., No.8, Zhonghua Road, Hsinchu Industrial Park, Hukou Township, Hsinchu County 30352, Taiwan; an_bang_duh@ccp.com.tw (A.-B.D.); ping_chieh_wang@ccp.com.tw (P.-C.W.)

**Keywords:** BPA type epoxy resin, hydrogenation, water as solvent, Rh catalyst, VulcanXC72, epoxy ring opening

## Abstract

A functional greener solvent mixture containing water, isopropyl alcohol (IPA) and ethyl acetate with the ratio 10:20:70 (wt%) was found to accelerate hydrogenation of bisphenol A type epoxy resin BE503 with a molecular weight of 1500 through an on-water mechanism, and led to an increased H_2_ availability, due to high solubility of H_2_ in IPA. Different carbon-based supports were tested and VulcanXC72 was found as the best support among the tested carbon-based ones as it possessed the highest amount of electron deficient promoter, RhO_x_. The catalyst, Rh_5_/VulcanXC72-polyol, synthesized by the microwave assisted polyol method, yielded a 100% hydrogenation of aromatic rings with an epoxy ring opening below 20.0% at 50 °C and a H_2_ pressure of 1000 psi in 2.25 h. Intrinsic activation energies for the hydrogenation of aromatic rings and epoxy ring opening were experimentally estimated and a mechanism for the hydrogenation of BE503 was proposed, wherein the hydrogenation of aromatic rings and epoxy ring opening in BE503 proceeded simultaneously in parallel and in-series with parallel being the major pathway.

## 1. Introduction

With the light emitting diode (LED) industry reported to have a current value of USD 15 billion and explosive growth predicted, research on light emitting diode (LED) packaging materials that can withstand yellowing at temperatures higher than 200 °C are in great demand. Bisphenol A type epoxy resins (BPAERs) are prime candidates for LED packaging as they are good insulators, highly durable and transparent. However, a rapid rise in temperature, coupled with radiation, causes epoxy resins to turn yellow and become susceptible to degradation by the oxidation of aromatic rings to produce benzoquinone.

Majorly, three proposed variants of BPAERs for a reduction in yellowing include the use of polysiloxane resins [1], BPAERs doped with UV-resistant additives and hydrogenated BPAERs (HBPAERs) [2,3,4]. While synthesizing silicone-based epoxy resins is complicated and involves expensive starting materials, BPAERs doped with UV-resistant additives are still susceptible to yellowing. Consequently, HBPAERs with easier synthesis and comparatively higher resistance to yellowing due to complete hydrogenation of aromatic rings are now being investigated with the purpose of being LED packaging materials [4]. In addition, the cost of HBPAERs is about 100 USD/kg, 30–50 times that of BPAERs. However, the epoxy rings in BPAERs are also highly reactive during hydrogenation of aromatic rings, leading to severe epoxy loss by C-O bond cleavage that needs to be avoided [5]. Preserving epoxy rings in HBPAERs is imperative as subsequent hardening of HBPAERs takes place by cross-linking of epoxy rings with hardeners.

Rhodium (Rh) is suggested to be used for selective hydrogenation of aromatic rings to reduce epoxy loss under mild reaction conditions [6]. Fung et al. [7] have reported the close to complete hydrogenation of BPAERs in tetrahydrofuran (THF) as the solvent using a Rh catalyst at 80 °C and 590 psi of H_2_ pressure for 3 h. However, Rh is expensive, and as a consequence, cheaper noble metals such as Pt or Ru are also employed, though they not as effective as Rh. The hydrogenation of a low molecular weight (MW) BPAER, BE186 with a MW of 373 and an epoxide equivalent weight (EEW) of 186 g/eq was previously studied by our group using a monometallic Rh and a bimetallic Rh-Pt catalyst, supported on carbon black VulcanXC72 synthesized by a microwave assisted polyol method [8]. The results showed that, though the bimetallic Rh-Pt catalyst exhibited higher catalytic activity than the monometallic Rh catalyst for the hydrogenation of aromatic rings in BE186, due to enhanced hydrogen (H_2_) spillover on the surface of Rh-Pt alloys, a higher epoxy loss was observed owing to the cleavage of C-O bond by Pt and thus Rh/VulcanXC72-polyol was preferred. A 100% hydrogenation yield of BE186 was reported using Rh/VulcanXC72-polyol at 60 °C and 1000 psi of H_2_ pressure for 3.5 h [8].

The presence of RhO_x_ in Rh/VulcanXC72-polyol acted as a strong electron deficiency (Lewis acidity) to promote the hydrogenation of aromatic ring in BE186, thus the support VulcanXC72 containing a moderate amount of oxygen groups was suggested [8]. A solvent mixture of water (H_2_O) in ethyl acetate (EA) was observed to help speed up the hydrogenation of BE186 due to the proton donation ability of functional solvent H_2_O, via a so called “on-water mechanism” that was also observed to be valid for the hydrogenation of bisphenol A in H_2_O using a Ru/MCM-41 catalyst [9,10,11].

Due to the low solubility of BPAERs in H_2_O, the hydrogenation of a high MW BPAER, BE503 (MW: 1500), called for an increased solubilization of solid BE503 in the solvent mixture. Isopropyl alcohol (IPA) was hence added into the H_2_O and EA solution as the ternary solvent in this study for the hydrogenation of BE186 and BE503 using a polyol synthesized Rh/VulcanXC72-polyol catalyst to propose a functionally superior and greener solvent mixture with higher H_2_O content to the previously reported binary 3 wt% H_2_O in EA mixture. The effects of different carbon-based supports on the activity of monometallic Rh catalyst for the hydrogenation of BE503 were also examined. The hydrogenation of BE503 was then desired to be 100% with an industrially acceptable epoxy ring opening of less than 20.0% by meticulously studying the influences of reaction variables including reaction temperature, pressure and time. Finally, based on an experimental estimation of the activation energies for the hydrogenation of aromatic rings and epoxy ring opening, a hydrogenation mechanism of BE503 was proposed.

## 2. Materials and Methods

### 2.1. Chemicals and Materials

Rhodium chloride hydrate (RhCl_3_.xH_2_O, Rh content: 38.5–45.5%), ethanol (EtOH, purity: 95.0%) and chloroform-d (CDCl_3_, purity: 99.8%) were purchased from Sigma-Aldrich (St. Louis, MO, USA). BPA type epoxy resins (BPAERs), BE186 and BE503 were provided by Chang Chun Plastics Co., Ltd. (Hukou, Taiwan, ROC). Details of the physical and chemical properties of BE186 and BE503 are shown in Table S1. BE186 is the diglycidyl ether of bisphenol A (DGEBA) and in liquid phase while BE503 is solid prepared using BE186 as the monomer. Figure S1 shows the representative chemical structure of a high MW BPAER synthesized via polymerization of BE186. Carbon black, VulcanXC72 was bought from Cabot Co., Ltd. (Boston, MA, USA). In addition, 1 M sodium hydroxide (NaOH, analytical grade) and 1 M hydrochloric acid (HCl, analytical grade) were purchased from Honeywell Fluka (Charlotte, NC, USA). Ethyl acetate (EA, purity: 99.5%) and methanol (MeOH, purity: 99.8%) were purchased from Macron Fine Chemicals (Radnor, PA, USA). Ethylene glycol (EG, purity: 99.0%) and isopropyl alcohol (IPA, purity: 99.5%) were purchased from J. T. Baker (Phillipsburg, NJ, USA). tert-Butanol (t-Butanol) (purity: 99.0%) was purchased from Alfa Aesar (Ward Hill, MA, USA). H_2_ (purity: > 99.9%) was purchased from Linde Lien Hwa Industrial Gases (Taipei, Taiwan, ROC). All chemicals and gases were used as received.

### 2.2. Catalyst Synthesis

All carbon supported Rh catalysts used in this study were synthesized using a microwave assisted polyol method previously reported by our group [8]. In brief, for a typical synthesis trial of a 5 wt% Rh catalyst supported on VulcanXC72, a calculated amount of Rh metal precursor (RhCl_3_.xH_2_O) was dissolved in 3:1 *v/v* of EG:H_2_O (120 mL). To this, 0.2 g of VulcanXC72 support was added and the pH of the slurry was controlled to 11–12 using 1 M NaOH. The alkaline slurry was then subjected to ultrasonication for 1.5 h to ensure uniform dispersion of the metal precursor on the surface of VulcanXC72. Following this, the slurry was heated twice, 30 s each using a microwave oven at 900 W. After the slurry was cooled down to room temperature, 1 M HCl was used to change its pH to 2–3, which allowed for the precipitation of Rh nanoparticles (NPs) on the surface of VulcanXC72. The catalyst powders were finally collected via suction filtration, washed several times with deionized (DI) water and allowed to completely dry overnight in an oven at 80 °C. The final catalyst was termed as Rh_5_/VulcanXC72-polyol, where 5 stood for the theoretical wt% loading of Rh.

### 2.3. Catalyst Characterization

N_2_ sorption isotherms were obtained on a Micromeritics ASAP 2060 autosorb (Norcross, GA, USA) and the Brunauer–Emmett–Teller (BET) method was used to measure surface area of the catalysts while the Barrett–Joyner–Halenda (BJH) method was used to measure the pore volume and pore diameter of the catalysts. All catalyst samples were degassed at 300 °C for 3 h prior to measurement. High resolution transmission electron microscopy (HRTEM) images were obtained using a JEOL JEM-F200 high resolution transmission electron microscope (Peabody, MA, USA). The catalyst samples for HRTEM were prepared on a 200-mesh lacey carbon-coated copper grid. To prepare the samples, a tiny amount of the catalyst powder was added to EtOH and subjected to ultrasonication long enough to ensure a high degree of suspension. After this, a single drop of the catalyst suspension was placed on the copper grid using a syringe needle and the gird was allowed to dry completely in a vacuum oven. Imaging software, ImageJ (Madison, WI, USA) was used to measure the average particle size of Rh and Rh-Pt alloy NPs by observing at least 100 particles in each of 5 representative TEM images. Wide angle X-ray diffraction (XRD) of Rh_5_/VulcanXC72-polyol was performed on a Bruker D8 Advance Eco X-ray powder diffractometer (Billerica, MA, USA). A Cu-Kα radiation of 40 kV, 20 mA was used with a scan speed of 0.5°/min for a 2θ range from 20°–80°. Particle size of the Rh_5_/VulcanXC72-polyol catalyst was measured using dynamic light scattering (DLS) performed on a Malvern Panalytical Zetasizer Nano ZS two angle particle and molecular size analyzer (Malvern, United Kingdom). X-ray photoelectron spectroscopy (XPS) was carried out on a ULVAC-PHI Quantera II X-ray photoelectron spectrometer with a +32-channel detector and 180° spherical capacitor (Kanagawa, Japan). Excitation was done using an Al anode X-ray source. The base vacuum pressure was less than 5 × 10^−5^ torr. Raman spectroscopy was performed on a Horiba Jobin Yvon (LABRAM HR 800 UV) Raman spectrometer (Kyoto, Japan).

### 2.4. Catalytic Activity Tests for Hydrogenation of BPAERs

The hydrogenation of the different BPAERs in this study was carried out in a 180-mL Thar stainless-steel reactor (Pittsburgh, PA, USA) equipped with a sapphire glass window in a semi-batch operation. A smaller glass vessel of 90 mL was placed in the reactor and all reactions were carried out inside this smaller glass vessel. An external magnetic stirrer was used for agitation and the smaller glass reaction vessel ensured that the magnetic stirrer was completely submerged in the liquid phase, thereby maintaining the uniform dispersion of the catalyst throughout the liquid solution. The reactor was equipped with a thermocouple that constantly monitored the inside temperature of the reactor and returned digital values of the inside reactor temperature through a digital display. In a typical reaction for the hydrogenation of BE503, 2 g of the reactant was added to 4.66 g of the solvent mixture and followed by the addition of 0.05 g of Rh_5_/VulcanXC72-polyol. The reactor was then purged at least three times with H_2_, followed by charging with H_2_ to a pressure of 1000 psi. The reactor was then heated up to the desired reaction temperature and held at the desired temperature for the duration of the reaction. A continuous flow of H_2_ was maintained to ensure there was no drop in H_2_ pressure. Unless stated otherwise, a stirring speed of 240 rpm was maintained. Once the reaction time was reached, the reactor was allowed to cool down to room temperature and was depressurized slowly. The reaction products were collected and thoroughly separated from the catalyst powders via centrifugation at 6000 rpm for 2 h. The solvent mixture was then slowly removed using rotary evaporation at 40 °C and the purified product was sent for subsequent analysis.

### 2.5. Analysis of Product for Hydrogenation Yield and Epoxy Ring Opening

Hydrogenation yield was the same as the extent of hydrogenation of aromatic rings (Yield%) and was measured using ^1^H NMR analysis (Bruker Avance 500, Billerica, MA, USA) with CDCl_3_ (δ: 7.26 ppm) as the d-solvent and calculated as the ratio of bisphenol A signal to aliphatic signal. Figure S2 depicts typical ChemDraw estimations of the ^1^H NMR spectra of BE503 (Figure S2a), completely hydrogenated BE503 (Figure S2b) and partially hydrogenated BE503 without and with epoxy ring (Figure S2c,d, respectively) indicating all the peaks along with an elaboration on the mathematical formula used for calculation of hydrogenation yield. The hydrogenation yield was completely independent of any epoxy ring opening that may have taken place during the reaction. This is shown through Figure S2d where the peaks assigned to H_2_ on aliphatic carbons after epoxy ring opening (h, i and j) did not overlap in any way with those used for calculating the extent of hydrogenation yield (i.e., H_2_ on unsaturated as well as saturated aromatic rings) and thus showed the mathematical formula used for the calculation of hydrogenation yield to be applicable even when epoxy ring opening took place. Epoxy ring opening (epoxy ring opening %) was measured with the help of Chang Chun Plastics Co., Ltd. (Hukou, Taiwan, ROC) in agreement with the guidelines laid out by the American Society for Testing and Materials (ASTM) for the standard test method for epoxy contents of epoxy resin (ASTM-D1652-11). To measure epoxy ring opening, HBPAERs were dissolved in an appropriate solvent and the solution was titrated in situ or using hydrogen bromide (HBr). Since epoxy groups directly react with HBr, epoxy ring opening was a function of the amount of acid consumed. For further details on the experimental procedure for measuring epoxy ring opening, the official ASTM-D1652-11 file may be looked into.

## 3. Results and Discussion

### 3.1. Solvent Screening for Hydrogenation of BE186 and BE503

For the hydrogenation of liquid-phase BE186 [8], a solvent mixture containing 3 wt% of H_2_O in EA (Solvent G) was found to further accelerate hydrogenation over Rh_5_/VulcanXC72-polyol through on-water mechanism [9,10,11,12]. Solvent G was chosen because it possesses a high α value (proton donation capability or the Bronsted acidity) and a low β value (proton acceptance capability or the Bronsted basicity) [8].

Since BE503 is solid, its hydrogenation becomes severely restricted by its limited solubility in the Solvent G. As such, the solvents with higher capability of solubilizing BPAERs should be used, in which a ternary alcohol, along with H_2_O and EA, was added in this study. The hydrogenation results of BE186 obtained using different solvent mixtures containing EA, water and various alcohol-based protic solvents in the same wt% ratio are reported in Table S2. A solvent mixture consisting of 3 wt% H_2_O, 7 wt% IPA and 90 wt% EA (Solvent 3, entry 4) was found to be more appropriate. The Kamlet–Taft table (Table S3) summarizes the solvatochromic parameters (α and β) of the tested alcohol-based solvents. The superiority of Solvent 3 stemmed from the fact that IPA had a sufficiently high α value and the highest H_2_ solubility among all the tested alcohol-based solvents (Table S3).

The effect of IPA amount on the solvent mixture for the hydrogenation of BE186 was investigated next and the results are presented in Table 1. Using Solvents 3, 5, 6 and 7 corresponding to an IPA ratio of 7, 27, 47 and 67 wt%, respectively (entries 1–4), Solvent 5 was found to be the most appropriate mixture. Although IPA increased the solubility of BPAERs and H_2_ in the solvent mixture, a higher IPA ratio adversely led to more pronounced competitive adsorption on the catalyst surface between the reactant and the solvent through proton acceptance, due to its large β value (Table S3). Following this, the wt% ratio of H_2_O in the solvent mixture was increased to 10 wt% to augment on-water mechanism. It was found that Solvent 8 (10 wt% H_2_O, 20 wt% IPA and 70 wt% EA) exhibited the highest BE186 hydrogenation yield of 65.9% (entry 5). When Solvent 8 was used for the hydrogenation of BE503, a hydrogenation yield of 70.8% was obtained. Using EA and solvent G for the hydrogenation of BE503 under the same reaction conditions, yields of only 54.2% and 64.0% were obtained, respectively, once again demonstrating the advantages of using IPA as a ternary solvent to increase reactant and H_2_ solubility in the solvent mixture.

### 3.2. Catalysts Characterization

All monometallic Rh catalysts were synthesized using a microwave assisted polyol method and their physicochemical properties were characterized using various techniques, including N_2_ sorption isotherm, high resolution transmission electron microscopy (HRTEM), wide angle X-ray diffraction (XRD), dynamic light scattering (DLS), X-ray photoelectron spectroscopy (XPS) and Raman spectroscopy.

**Figure 1:**
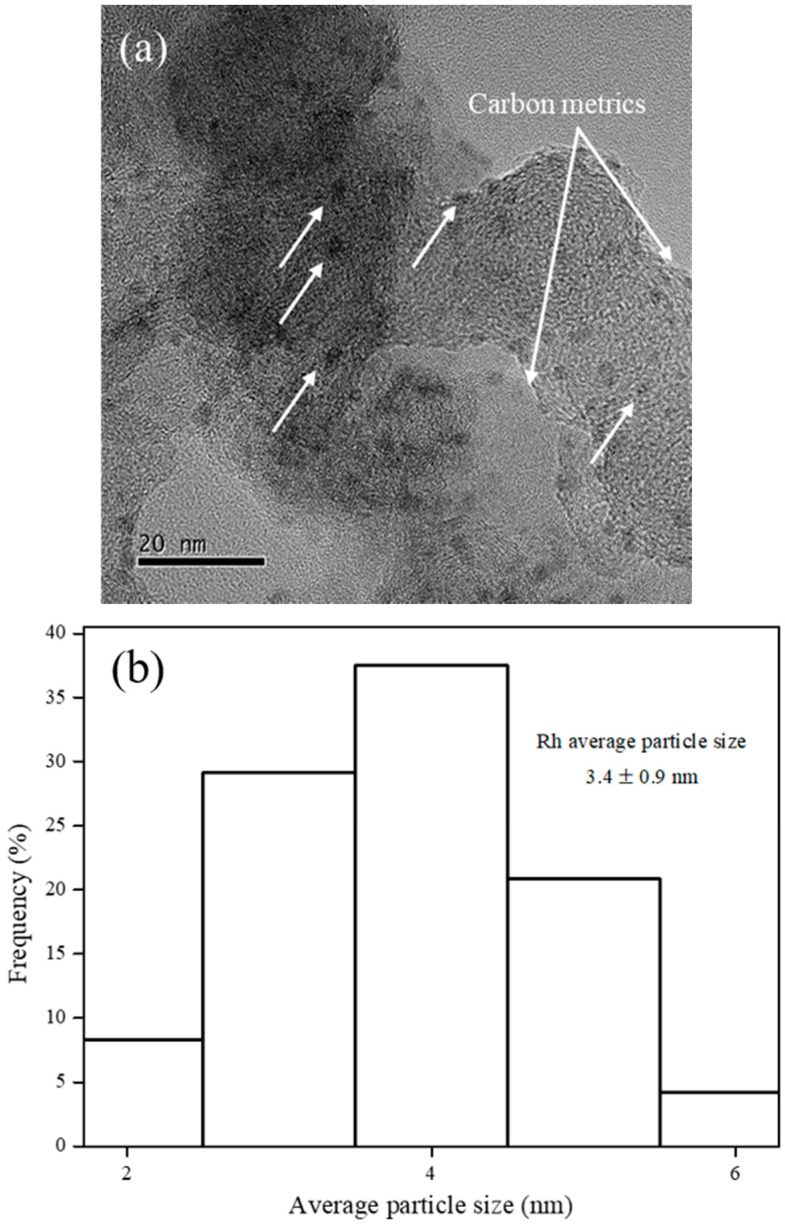

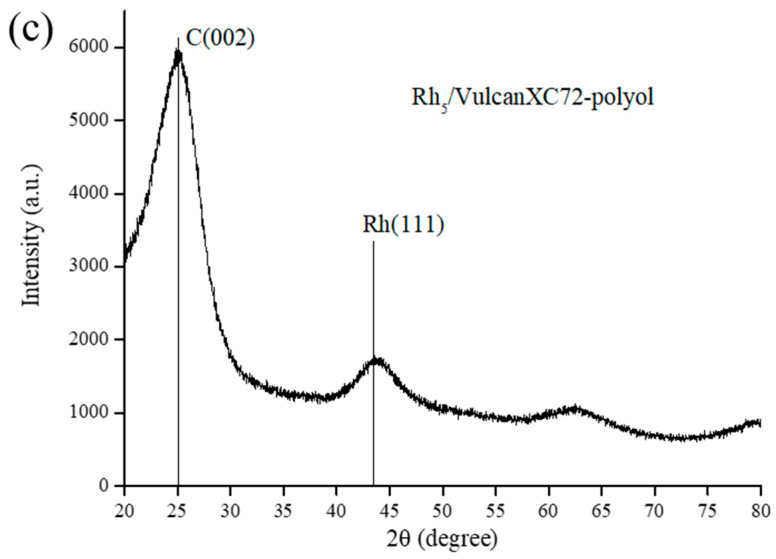
Morphology of Rh_5_/VulcanXC72-polyol as its (**a**) HRTEM image with Rh NPs located on the outer surface of the catalyst as indicated by the white arrows, (**b**) corresponding histograms of average particle size analysis and (**c**) X-ray diffractogram.

N_2_ sorption isotherm of VulcanXC72 revealed a type IV hysteresis loop, typical of mesoporous materials [8]. Virgin VulcanXC72 possessed a BET surface area of 232.8 m^2^/g with a pore diameter and pore volume of 13.6 nm and 0.47 cm^3^/g, respectively. After doping with Rh, the BET surface area of Rh_5_/VulcanXC72-polyol reduced slightly by 4.1% to 223.3 m^2^/g. The negligible reduction in surface area of VulcanXC72 indicated that the majority of Rh nanoparticles (NPs) were located on the outer surface of VulcanXC72, thus inhibiting severe pore blockage. The HRTEM image of Rh_5_/VulcanXC72-polyol shown in Figure 1a further confirms that Rh was located majorly on the outer surface of the catalyst, as indicated by the white arrows. Rh_5_/VulcanXC72-polyol possessed a Rh particle size of 3.4 ± 0.9 nm, as calculated from its HRTEM image (Figure 1b). It was also speculated that the rapidness of the polyol synthesis method might have played a part in ensuring that the location of Rh was mostly on the outer surface of VulcanXC72 by not allowing enough time for the Rh NPs to diffuse and deposit themselves inside the pores of VulcanXC72. XRD analysis of Rh_5_/VulcanXC72-polyol was also performed and the corresponding X-ray diffractogram can be seen in Figure 1c. The X-ray diffractograms of Rh_5_/VulcanXC72-polyol revealed broad and not very discernible peaks for Rh (111) and was attributed to the small size and good dispersion of Rh NPs obtained due to polyol synthesis.

A number of disordered carbon metrics emerged on the surface of the active sites of the catalyst as a result of a strong coordination between the oxygen groups on the surface of VulcanXC72 and Rh NPs, leading to the emergence of RhO_x_ (Figure 1a), which acted as a promoter for the hydrogenation of BPAERs and was crucial to the high aromatic ring hydrogenation ability of Rh_5_/VulcanXC72-polyol [8]. DLS was used to measure the average catalyst particle diameter of Rh_5_/VulcanXC72-polyol and a Z-average value of 1.92 × 10^−6^ m was reported.

**Figure 2:**
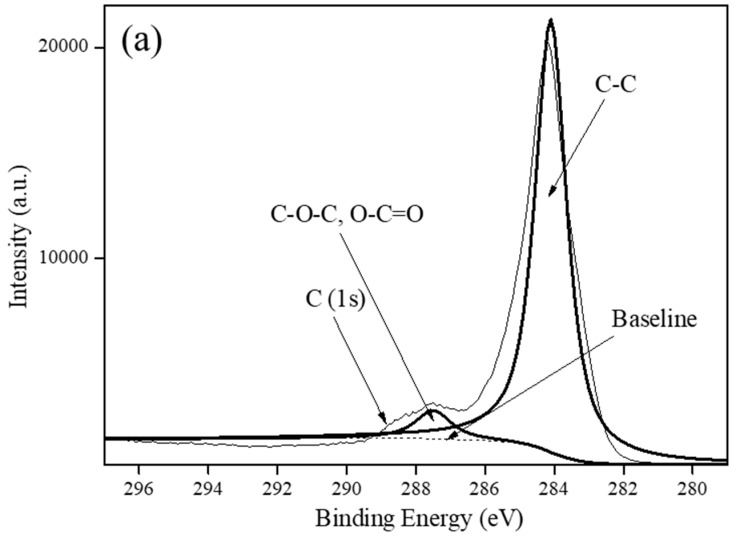

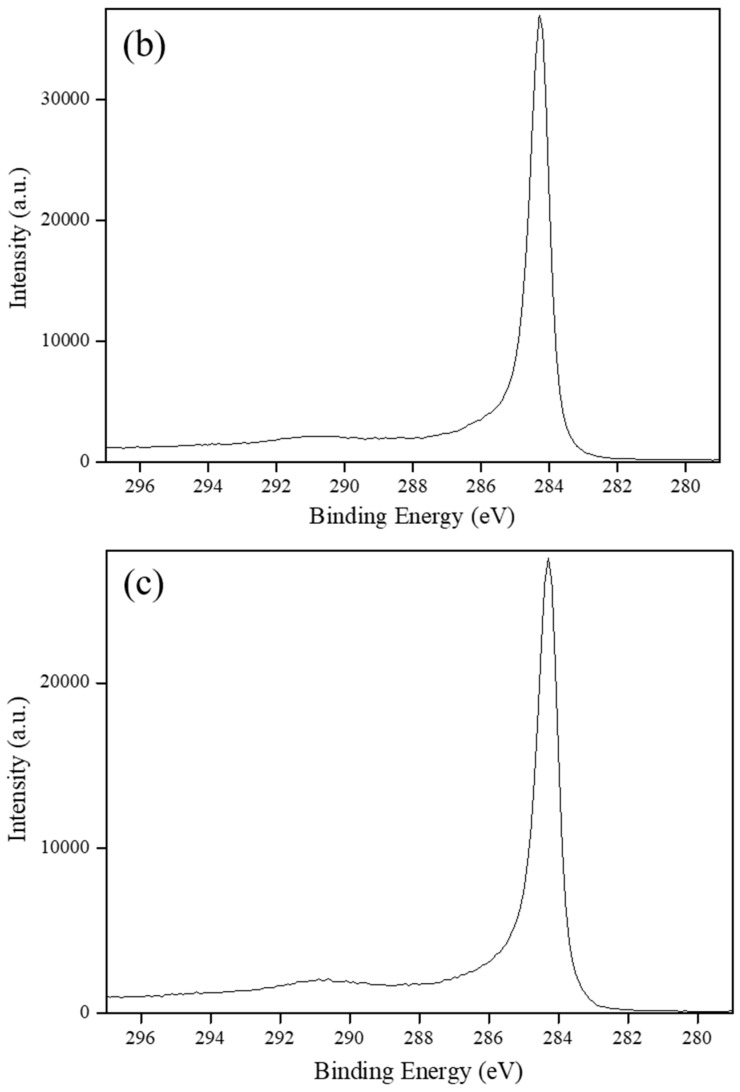
XPS spectra of: (**a**) VulcanXC72, (**b**) Graphene and (**c**) MWCNTs.

Carbon-based supports, including VulcanXC72, graphene and MWCNTs, were employed as the support materials to synthesize Rh_5_/VulcanXC72-polyol and XPS, in conjunction with Raman spectroscopy, helped elucidate the effect of the catalyst supports on the hydrogenation of BE503. The XPS spectra of the three carbon-based supports in the C (1s) region are shown in Figure 2. Based on a deconvolution of the XPS spectrum of VulcanXC72, a binding energy (BE) of 284.1 eV was attributed to the C–C component of VulcanXC72, while a BE of 287.5 eV was attributed to the coordination of oxygen group with carbon on the surface of VulcanXC72 as ester (C–O–), carbonyl (C=O) and carbonate (C=O) (Figure 2a) [8]. Graphene and MWCNTs showed no such BE at 287.5 eV and only furnished a BE of 284.3 eV, attributed to the C-C component (Figure 2b,c, respectively). From XPS, VulcanXC72 possessed 6.9% oxygen groups (atom%), which was close to the reported value of 7.8% [13].

Raman spectra (Figure S3) revealed the degree of graphitization in the supports as a function of the intensity ratio of the G band (I_g_) to D band (I_d_) in the order: graphene (I_g_/I_d_ = 2.652) > MWCNT (I_g_/I_d_ = 1.012) > VulcanXC72 (I_g_/I_d_ = 1.097). A higher I_g_/I_d_ ratio of graphene indicated that graphene possessed mostly six membered carbon rings with sp^2^ hybridization. On the other hand, much lower I_g_/I_d_ ratios of VulcanXC72 and MWCNTs suggested that VulcanXC72 and MWCNTs were primarily made up of five or seven membered carbon rings with sp^3^ hybridization [14].

**Figure 3:**
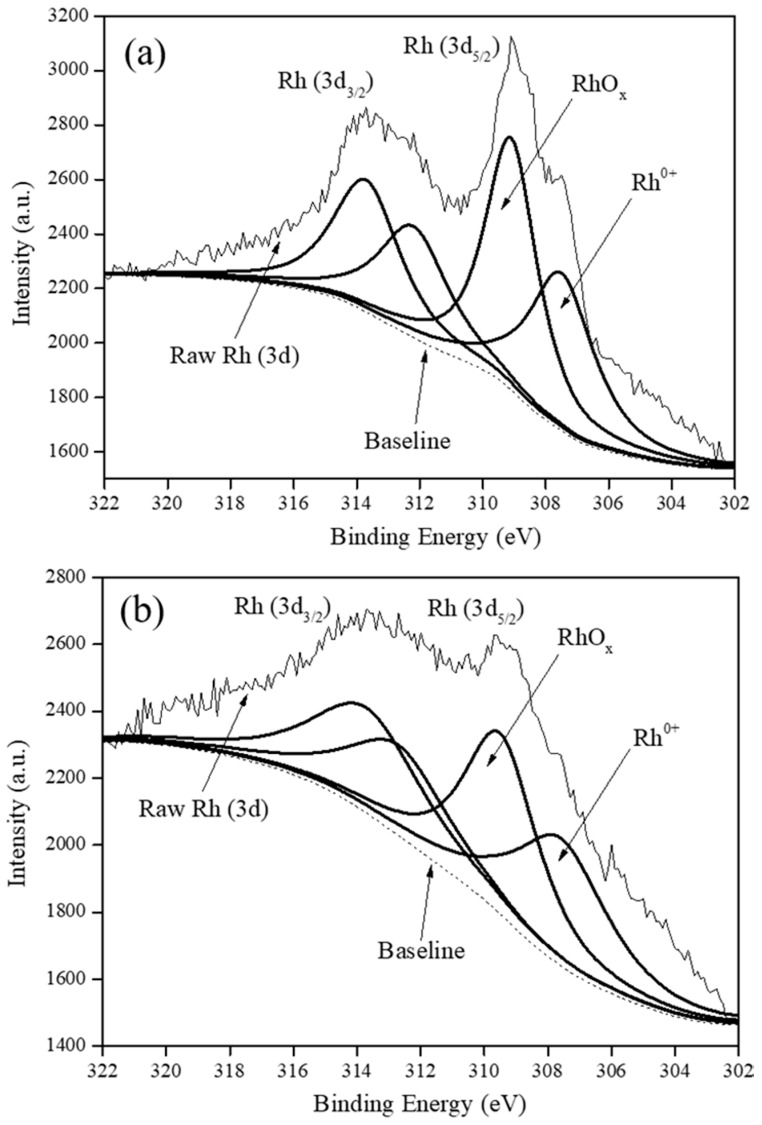

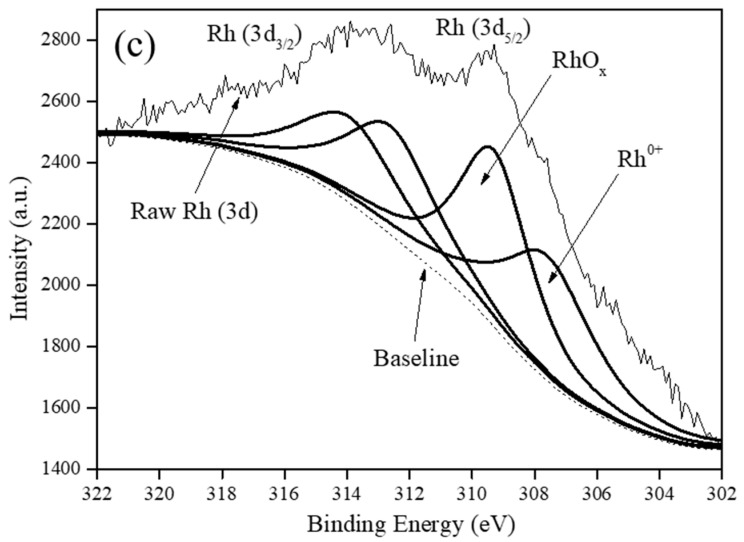
XPS spectra of: (**a**) Rh_5_/VulcanXC72-polyol, (**b**) Rh_5_/Graphene-polyol and (**c**) Rh_5_/MWCNTs-polyol.

Figure 3 shows the deconvoluted XPS spectra of all the various carbon supported Rh catalysts in the Rh (3d_5/2_) and Rh (3d_3/2_) regions with BEs from 302.0–311.9 eV and 312.0–320.0 eV, respectively [8,15].

It was understood that in the Rh (3d_5/2_) region, Rh existed in at least three oxidation states in all the catalysts as Rh^0^ (BE of 307.9 eV), Rh^1+^ (BE of 308.0–308.9 eV) and Rh^3+^ (BE range of 309.0–311.9 eV) [8]. Although, graphene and MWCNTs were devoid of any oxygen groups, Rh_5_/Graphene-polyol and Rh_5_/MWCNTs-polyol still contained RhO_x_ attributed to incomplete reduction during the rapid polyol synthesis method. A quantification of the RhO_x_ content in the catalysts using XPS is presented in Table S4 and reveals that Rh_5_/VulcanXC72-polyol possessed the highest RhO_x_ content at 55.9% (atom%).

### 3.3. Hydrogenation of Higher MW BPAER, BE503

#### 3.3.1. Effects of Different Carbon-Based Supports

The hydrogenation of BE503 in Solvent 8 was performed over Rh_5_/VulcanXC72-polyol, Rh_5_/Graphene-polyol and Rh_5_/MWCNTs-polyol at 40 °C and a H_2_ pressure of 1000 psi for 2 h to choose the best support. The activities of the different catalysts in terms of hydrogenation yields were found to vary in the order: Rh_5_/VulcanXC72-polyol (70.8%) > Rh_5_/MWCNTs-polyol (44.7%) > Rh_5_/Graphene-polyol (25.3%). The difference in the hydrogenation yields between Rh_5_/VulcanXC72-polyol and Rh_5_/MWCNTs-polyol, as high as 26.1%, was directly attributed to Rh_5_/VulcanXC72-polyol possessing the highest RhO_x_ that assisted in activating aromatic rings, making them more susceptible for hydrogenation. Factors, such as hydrophilicity of VulcanXC72 over hydrophobic graphene and MWCNTs, that enhanced the dispersion of VulcanXC72 in the hydrophilic solution during catalyst synthesis [16] and stronger metal-support interactions in Rh_5_/VulcanXC72-polyol due to strong coordination between the oxygen groups of VulcanXC72 and Rh NPs, may have further enhanced the activity of Rh_5_/VulcanXC72 polyol.

From the Raman spectra, it was understood that, since VulcanXC72 and MWCNTs were made up mostly of five or seven-membered carbon rings, Rh_5_/VulcanXC72-polyol and Rh_5_/MWCNTs-polyol possessed greater tension than Rh_5_/Graphene-polyol with six-member carbon rings and were, therefore, structurally weaker and more reactive as an easy link up of carbon atoms with H atoms made them efficient H atom carriers.

The presence of a large number of oxygen groups on the support surface (>10.0%), on the other hand, would adversely affect the properties of hydrogenated BE503 by causing an increase in epoxy ring opening, as was seen previously for BE186 [8]. Since VulcanXC72 possessed only 6.9% oxygen groups, Rh_5_/VulcanXC72-polyol was therefore endorsed as an effective catalyst for the hydrogenation of BE503 with proper control of epoxy ring opening.

#### 3.3.2. Effects of Reaction Parameters

To achieve 100% hydrogenation of aromatic ring in BE503 with effective control of the epoxy ring opening, the reaction conditions were varied using Rh_5_/VulcanXC72-polyol in Solvent 8. Since BE503 possessed a higher number of epoxy rings than BE186, a maximum epoxy ring opening of 20.0% was predetermined as an industrial standard, at which the subsequent HBPAER hardening process would not be adversely affected. The obtained results for the hydrogenation of BE503 with different operation variables, including temperature, H_2_ pressure and time, are presented in Table 2.

As can be seen from Table 2, a 100% hydrogenation yield could be achieved in 2 h at a H_2_ pressure of 1000 psi and a temperature of 60 °C. However, under these reaction conditions, the epoxy ring opening was 29.3% compared to the 20.0% limit (entry 3). When the temperature was reduced to 50 °C and time was increased to 2.25 h at a H_2_ pressure of 1000 psi, a 100% hydrogenation yield of BE503 with 19.3% epoxy ring opening could be achieved (entry 4). However, if the reaction times were further shortened to 1.5 h and 1.25 h at a temperature of 60 °C and a H_2_ pressure of 1000 psi, although 100% hydrogenation yields were still achieved, the epoxy ring openings were still more than 5.0% above the permissible limit (entries 5 and 6). If the reaction time was reduced to 1 h, only 90.7% hydrogenation yield with 21.3% epoxy ring opening was observed (entry 7), but when the reaction time was increased to 2 h, the epoxy ring opening was 29.3% (entry 3). The obtained results indicated that epoxy ring opening was directly proportional to reaction time and continued occurring even after complete hydrogenation of aromatic rings. Therefore, it was pertinent to find a set of reaction conditions at which a 100% hydrogenation yield coincided with the end of the reaction to ensure epoxy ring opening could be kept below 20.0%. It can be seen from entries 8 and 9 that, even at a shorter reaction time of 1.5 h, if the reaction temperature exceeded 50 °C, the epoxy ring opening would subsequently increase beyond 20.0%. It could then be concluded that 50 °C was the maximum allowable reaction temperature for the hydrogenation of BE503 using Rh_5_/VulcanXC72-polyol. Gradually decreasing the H_2_ pressure from 1000 psi to 600 psi with all other reaction conditions kept the same led to a gradual decrease in epoxy ring opening, thus revealing the direct dependency of epoxy ring opening to H_2_ pressure (entries 5, 10 and 11).

It might be concluded from Table 2 that the effects of operation variables on epoxy ring opening varied in the order temperature > time > H_2_ pressure. The operation at 50 °C and a H_2_ pressure of 1000 psi for 2.25 h (entry 4) was therefore suggested, at which a 100% hydrogenation yield with epoxy ring opening below 20.0% could be achieved.

### 3.4. Kinetics of Hydrogenation of Aromatic Ring and Epoxy Ring Opening in BE503

Activation energies for the hydrogenation of aromatic rings and epoxy ring opening in BE503 were measured by assuming a pseudo first order reaction with respect to BE503. The kinetics were measured by confirming that the hydrogenation of BE503 took place without intraparticle diffusion and interphase mass transfer resistances. For the reaction to proceed without any intraparticle diffusion resistance, the Weisz–Prater criteria at different reaction conditions were ascertained to be less than 1.0 as shown in Table S5 [17]. Interphase mass transfer resistance was observed to be neglected, since the hydrogenation of BE503 at two stirring speeds of 60 and 240 rpm under the reaction conditions of 55 °C and H_2_ pressure of 1000 psi for 1.25 h using Solvent 8 gave similar hydrogenation yields.

**Figure 4:**
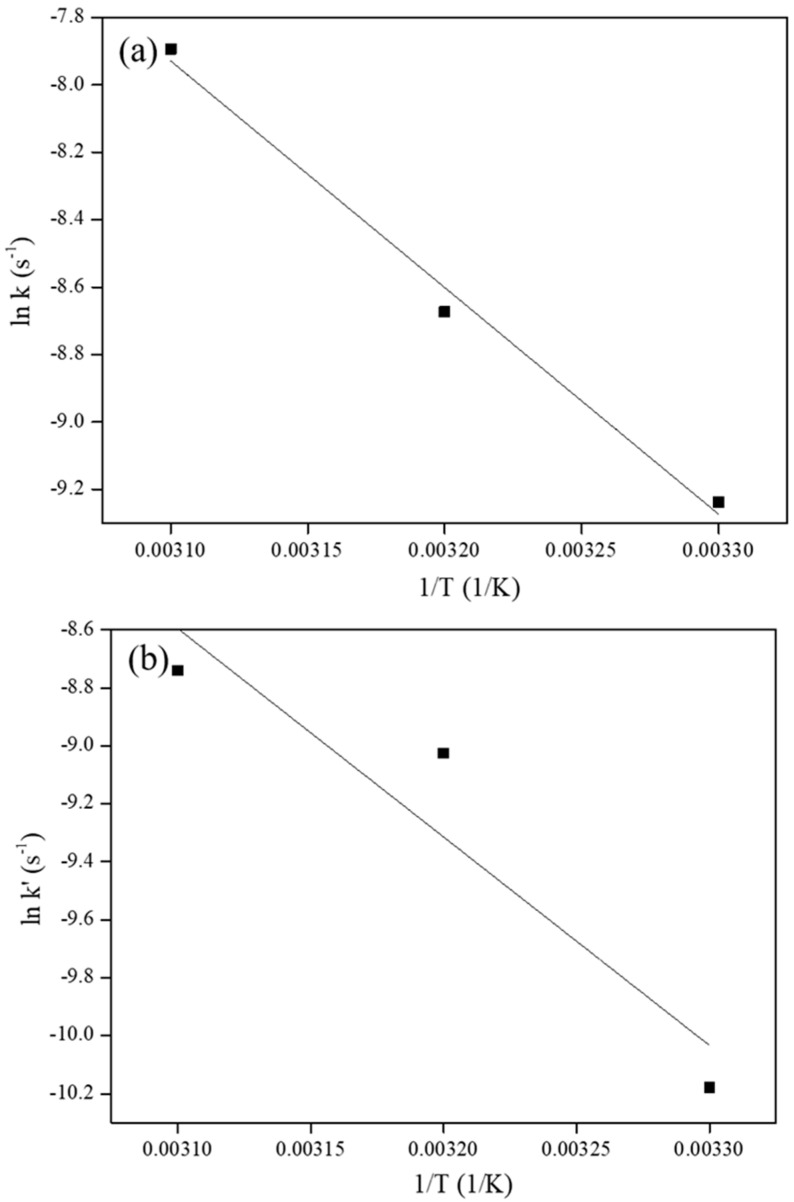
Plot of (**a**) ln *k* vs. 1/T (for hydrogenation yield) and (**b**) ln *k’* vs. 1/T (for epoxy ring opening).

Two measured pseudo first order rate constants, *k* (s^−1^), for the hydrogenation of aromatic rings and *k’* (s^−1^), for epoxy ring opening at three reaction temperatures of 30, 40 and 50 °C and the subsequent calculation of the activation energies for the hydrogenation of aromatic rings and epoxy ring opening in BE503 are shown in Table 3. A linear increase in the values of the observed rate constant with respect to temperature indicated that hydrogenation of BE503 and epoxy ring opening indeed follow pseudo first order kinetics. Plots of *ln k* vs. *1/T* and (*ln k’*) vs. (*1/T*) are depicted in Figure 4. The estimated activation energies for the hydrogenation of aromatic rings and epoxy ring opening, and the corresponding Arrhenius Equations (1) and (2), are as follows:

(1) $$\ln\left(k\right)=\;-54563/\left[8.314\;\left(\frac{J}{mol\times K}\right)\times \;T\;\left(K\right)\right]\;+\;12.381$$

(2) $$\ln\left(k{}^{\prime }\right)=\;-58853/\left[8.314\;\left(\frac{J}{mol\times K}\right)\times \;T\;\left(K\right)\right]\;+\;13.316$$

While the activation energy for hydrogenation of aromatic rings (54,563 J/mol) was found to be lower than that for epoxy ring opening (58,853 J/mol), the close proximity of the two values indicated that Rh_5_/VulcanXC72-polyol catalyzed the hydrogenation of aromatic rings parallel to epoxy ring opening, as can be confirmed from Table 2, where even the maximum epoxy ring opening of 29.3% took place alongside the hydrogenation of aromatic rings. It is also seen in Table 2 that, even if 100% hydrogenation yield was achieved, epoxy ring opening kept on increasing further as an in-series reaction, similar to the in-series hydrogenation of bis (benzene) ring compounds [18].

## 4. Conclusions

The hydrogenation of high MW BPAER, BE503 with a MW of 1500 under mild reaction conditions was performed in this study from the aspects of solvent selection, choice of catalyst support and modification to operation variables to maximize hydrogenation yield to 100% while maintaining epoxy ring opening below 20.0%. A greener ternary solvent mixture consisting of 10 wt% H_2_O, 20 wt% IPA and 70 wt% EA denoted as Solvent 8 was most effective for the hydrogenation of BE186 and BE503 using Rh_5_/VulcanXC72-polyol through on-water mechanism and higher solubilities of BPAER and H_2_ because of the presence of IPA in the liquid mixture. Using carbon-based supports, VulcanXC72, graphene and MWCNTs, Rh_5_/VulcanXC72-polyol emerged superior for the hydrogenation of BE503, owing to its possessing the appropriate number of oxygen groups and subsequently the highest number of RhO_x_. VulcanXC72 also acted as an efficient H atom carrier in reaction, as the carbon atoms in Rh_5_/VulcanXC72-polyol could link-up easily with H atoms. In total, 100% hydrogenation yield of BE503 with epoxy ring opening below 20.0% was achieved at 50 °C, and 1000 psi of H_2_ pressure for 2.25 h of reaction was achieved, which was lower by 10 °C and faster by 1.25 h than what was reported for lower MW BE186 using Solvent G. Similarly, when compared to a previously reported study on the hydrogenation of BPAERs using a Rh catalyst and THF as the solvent at 80 °C for 3 h with 99.5% hydrogenation yield, the complete hydrogenation of BE503 reported in this study was milder by 30 °C and faster by 0.75 h.

The activation energy for the hydrogenation of BE503 using Rh_5_/VulcanXC72-polyol was experimentally measured to be 54,563 J/mol while that for epoxy ring opening was 58,853 J/mol. From the activation energies and reaction data obtained at different reaction conditions, it was concluded that the hydrogenation of aromatic rings and epoxy ring opening in BE503 took place as simultaneous parallel and in-series reactions with the parallel pathway being the dominant one.

## Figures and Tables

**Table 1:** Hydrogenation of BE186 and BE503 using different ratios of IPA (wt%) in solvent mixtures.

Entry	Solvent	Concentration (wt%)	Time (h)	Yield (%)
1 *	Solvent 3 (3 wt% H_2_O, 7 wt% IPA, 90 wt% EA)	50	0.5	62.4
2 *	Solvent 5 (3 wt% H_2_O, 27 wt% IPA, 70 wt% EA)	50	0.5	63.0
3 *	Solvent 6 (3 wt% H_2_O, 47 wt% IPA, 50 wt% EA)	50	0.5	59.7
4 *	Solvent 7 (3 wt% H_2_O, 67 wt% IPA, 30 wt% EA)	50	0.5	58.2
5 *	Solvent 8 (10 wt% H_2_O, 20 wt% IPA, 70 wt% EA)	50	0.5	65.9
6 **	Solvent 8 (10 wt% H_2_O, 20 wt% IPA, 70 wt% EA)	30	2	70.8
7 **	EA	30	2	54.2
8 **	Solvent G (3 wt% H_2_O, 97 wt% EA)	30	2	64.0

Note: 2 g BPAER (* BE186, ** BE503), 2 g solvent for entries 1–5, 4.66 g solvent for entries 6–8, 0.05 g Rh_5_/VulcanXC72-polyol, H_2_ pressure of 1000 psi, 40 °C, concentration: W_reactant_/W_reactant+solvent_, RSD for hydrogenation yield ≤ 2%.

**Table 2:** Hydrogenation of BE503 using Rh_5_/VulcanXC72-polyol at different reaction conditions.

Entry	Temperature (℃)	Pressure (psi)	Time (h)	Yield (%)	Epoxy Ring Opening (%)
1	40	1000	2	70.8	18.9
2	50	1000	2	93.2	18.6
3	60	1000	2	100	29.3
4	50	1000	2.25	100	19.3
5	60	1000	1.5	100	25.9
6	60	1000	1.25	100	25.4
7	60	1000	1	90.7	21.3
8	55	1000	1.5	100	24.8
9	52	1000	1.5	89.4	22.3
10	60	800	1.5	100	24.7
11	60	600	1.5	98.2	21.8

Note: 2 g BE503, 4.66 g solvent 8, 0.05 g Rh_5_/VulcanXC72-polyol, concentration: W_reactant_/W_reactant+solvent_ (30 wt%), RSD for hydrogenation yield ≤ 2%.

**Table 3:** Calculated pseudo first order rate constants and activation energies for the hydrogenation of BE503 and epoxy ring opening using Rh_5_/VulcanXC72-polyol.

Entry	Temperature (℃)	Yield (%)	*k* (s^−1^)	Activation Energy (Hydrogenation) (J/mol)	Epoxy Ring Opening (%)	*k’* (s^−1^)	Activation Energy, (Epoxy Ring Opening) (J/mol)
1	30 °C	50.4	9.74 × 10^−5^	54,563	13.9	3.80 × 10^−5^	58,853
2	40 °C	70.8	1.71 × 10^−4^		18.9	1.20 × 10^−4^	
3	50 °C	93.2	3.73 × 10^−4^		18.6	1.60 × 10^−4^	

Note: 2 g BE503, 4.66 g solvent 8, 0.05 g Rh_5_/VulcanXC72-polyol, H_2_ pressure of 1000 psi for 2 h, concentration: W_reactant_/W_reactant + solvent_ (30 wt%), RSD for hydrogenation yield ≤ 2%.

## Supplementary Materials

The following are available online at https://www.mdpi.com/2073-4360/12/11/2513/s1, Table S1: Physical and chemical properties of BPAERs, Table S2: Hydrogenation of BE186 using different protic alcohol-based solvents in solvent mixtures, Table S3: Kamlet–Taft table for common solvents (including green solvents) and alcohol-based solvents for hydrogenation of BE186 plus the solubility of H_2_ in the alcohol-based solvents, Table S4: XPS deconvolution results of monometallic Rh catalysts supported on different carbon-based supports in the Rh (3d_5/2_) region, Table S5: Measured values of Weisz–Prater criteria for the hydrogenation of BE503 using Rh_5_/VulcanXC72-polyol at different temperatures, Figure S1: Representative chemical structure of a high MW BPAER synthesized via polymerization of BE186, Figure S2: Typical estimations of the ^1^H NMR spectra of (a) BE503, (b) completely hydrogenated BE503, (c) partially hydrogenated BE503 with calculation for hydrogenation yield and (d) partially hydrogenated BE503 with epoxy ring opening, Figure S3: Raman spectra of (a) VulcanXC72, (b) Graphene and (c) MWCNTs.
