# Supplementary material for: Hydrogenation of High Molecular Weight Bisphenol A Type Epoxy Resin BE503 in a Functional and Greener Solvent Mixture Using a Rh Catalyst Supported on Carbon Black
Source: Polymers (Basel). 2020 Oct 28;12(11):2513. doi: 10.3390/polym12112513 (PMC7693935; doi:10.3390/polym12112513)
Supplement: Supplementary file 1 [file polymers-12-02513-s001.pdf]

polymers

Supplementary Materials

Hydrogenation of High Molecular Weight Bisphenol A Type Epoxy Resin BE503 in a Functional and Greener Solvent Mixture using a Rh Catalyst Supported on Carbon Black

Bo-Xin Lai[1], Saurav Bhattacharjee[1], Yi-Hao Huang[1], An-Bang Duh[2], Ping-Chieh Wang[2] and Chung-Sung Tan[1,*]

[1] Department of Chemical Engineering, National Tsing Hua University, No. 101, Section 2, Guangfu Road, East District, Hsinchu City, 30013, Taiwan, ROC; s105032892@m105-mail.nthu.edu.tw
[2] Chang Chun Plastics Co., Ltd., No.8, Zhonghua Road, Hsinchu Industrial Park, Hukou Township, Hsinchu County, 30352, Taiwan, ROC; an_bang_duh@ccp.com.tw, ping_chieh_wang@ccp.com.tw
* Correspondence: cstan@mx.nthu.edu.tw; Tel.: +886-3-572-1189

Received: date; Accepted: date; Published: date

This supplementary material file contains:

Total number of pages: 12 (1-12)

Total number of tables: 5 (S1-S5)

Total number of figures: 3 (S1-S3)

Table S1. Physical and chemical properties of BPAERs.

**Property**	**BPAER**	
	**BE186**	**BE503**
Molecular weight (MW)	373	1500
Viscosity (cp)	11620	n/a
EEW[a] (g/eq)	186	751
Appearance	liquid	solid
Color (gardner[b])	transparent	0.2
Softening point[c] (°C)	n/a	94.8

a: epoxide equivalent weight (EEW) defined as the weight of BPAER containing 1 mole of epoxy group, b: a one-dimensional scale used to measure extent of yellowness, c: the temperature at which a solid material softens to the extent that it starts dripping, n/a: not applicable.

Table S2. Hydrogenation of BE186 using different protic alcohol-based solvents in solvent mixtures.

Entry	Solvent	Yield (%)
1	Solvent G (3 wt% H_2O, 97 wt% EA)	59.6
2	Solvent 1 (3 wt% H_2O, 7 wt% MeOH, 90 wt% EA)	57.9
3	Solvent 2 (3 wt% H_2O, 7 wt% EtOH, 90 wt% EA)	55.3
4	Solvent 3 (3 wt% H_2O, 7 wt% IPA, 90 wt% EA)	62.4
5	Solvent 4 (3 wt% H_2O, 7 wt% t-Butanol, 90 wt% EA)	59.5

2 g BE186, 2 g solvent, 0.05 g Rh_5/VulcanXC72-polyol, H_2 pressure of 1000 psi, 40 ^oC for 0.5 h, concentration: $W_{reactant}/W_{reactant+solvent}$ (50 wt%), RSD for hydrogenation yield ≤ 2%.

Table S3. Kamlet-Taft table for common solvents (including green solvents) and alcohol-based solvents for hydrogenation of BE186 plus the solubility of H_2 in the alcohol-based solvents.

**Solvent**	**π (Polarity index)**	**α (Hydrogen bonding donor)**	**β (Hydrogen bonding acceptor)**	**H_2 solubility (Mole fraction at 25 °C, 1 atm) (X_{H_2} *10^4)**
H_2O	1.09	1.17	0.18	--
EA	0.55	--	0.45	--
MeOH	0.60	0.93	0.62	1.61
EtOH	0.54	0.83	0.77	2.06
IPA	0.48	0.76	0.95	4.61
t-Butanol	0.41	0.68	1.01	3.28

Reprinted (adapted) with permission from Ref. [1]. Copyright 1983 American Chemical Society.

Table S4. XPS deconvolution results of monometallic Rh catalysts supported on different carbon-based supports in the Rh ($3d_{5/2}$) region.

**Catalyst**	**RhO_x (BE, eV)**	**RhO_x (atom %)**	**Rh^{0+} (BE, eV)**	**Rh^0 (atom %)**	**RhO_x/ Rh^0**
Rh_5/VulcanXC72-polyol	309.1	55.9	307.5	44.1	1.27
Rh_5/Graphene-polyol	309.5	52.8	307.6	47.2	1.12
Rh_5/MWCNTs-polyol	309.3	52.7	307.6	47.3	1.11

Table S5. Measured values of Weisz-Prater criteria for the hydrogenation of BE503 using Rh_5/VulcanXC72-polyol at different temperatures.

Temperature (°C)	D	D_e	C_{w-p}
30	3.26×10^{-6}	$2.91*10^{-7}$	$3.08*10^{-10}$
40	$3.37*10^{-6}$	$3.01*10^{-7}$	$5.24*10^{-10}$
50	$3.48*10^{-6}$	$3.10*10^{-7}$	$1.11*10^{-9}$

D = diffusion coefficient, D_e: effective diffusivity, C_{w-p}: Weisz-Prater criteria*

$$^{*}C_{w-p} = \frac{r_A(d_p/2)^2}{C_{A,S}\, D_e}$$

Where: $D_e = D\,\frac{\varepsilon}{\tau}$

$$D = 7.4*10^{-12}\frac{T\sqrt{\chi M}}{\mu_1\, \nu^{0.6}}$$

χ (association factor of solvent) = 1

M (molecular weight of the solvent) = 0.088 kg/mol (EA is major solvent in Solvent 8)

μ_1 (viscosity of the solution) = $11.5*10^{-3}$ kg/m*s

ν (molecular volume of the reactant) = molecular weight of reactant/ density of reactant = 1.5/1244 = $1.206*10^{-3}$ m^3/mol

ε (support porosity) = $V_{pore}/V_{support}$ = 0.467*0.097/0.127 = 0.357

τ (tortuosity factor) = 4 [2]

$C_{A,S}$ (surface concentration of the reactant) = $1.96*10^{2}$ mol/m^3

d_p (catalyst particle diameter) = $1.92*10^{-6}$ m

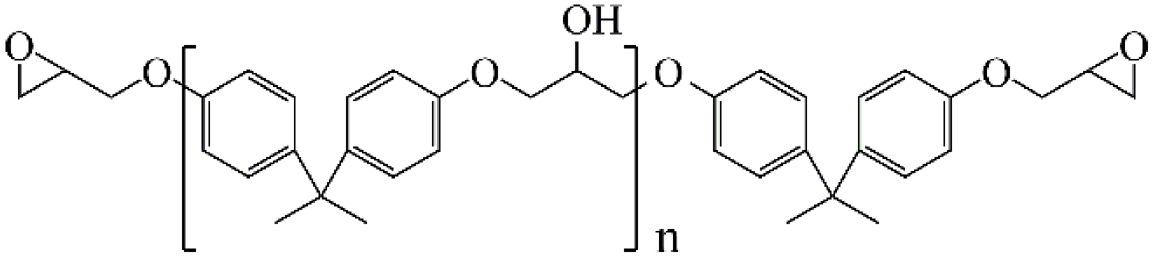

Figure S1. Representative chemical structure of a high MW BPAER synthesized via polymerization of BE186.

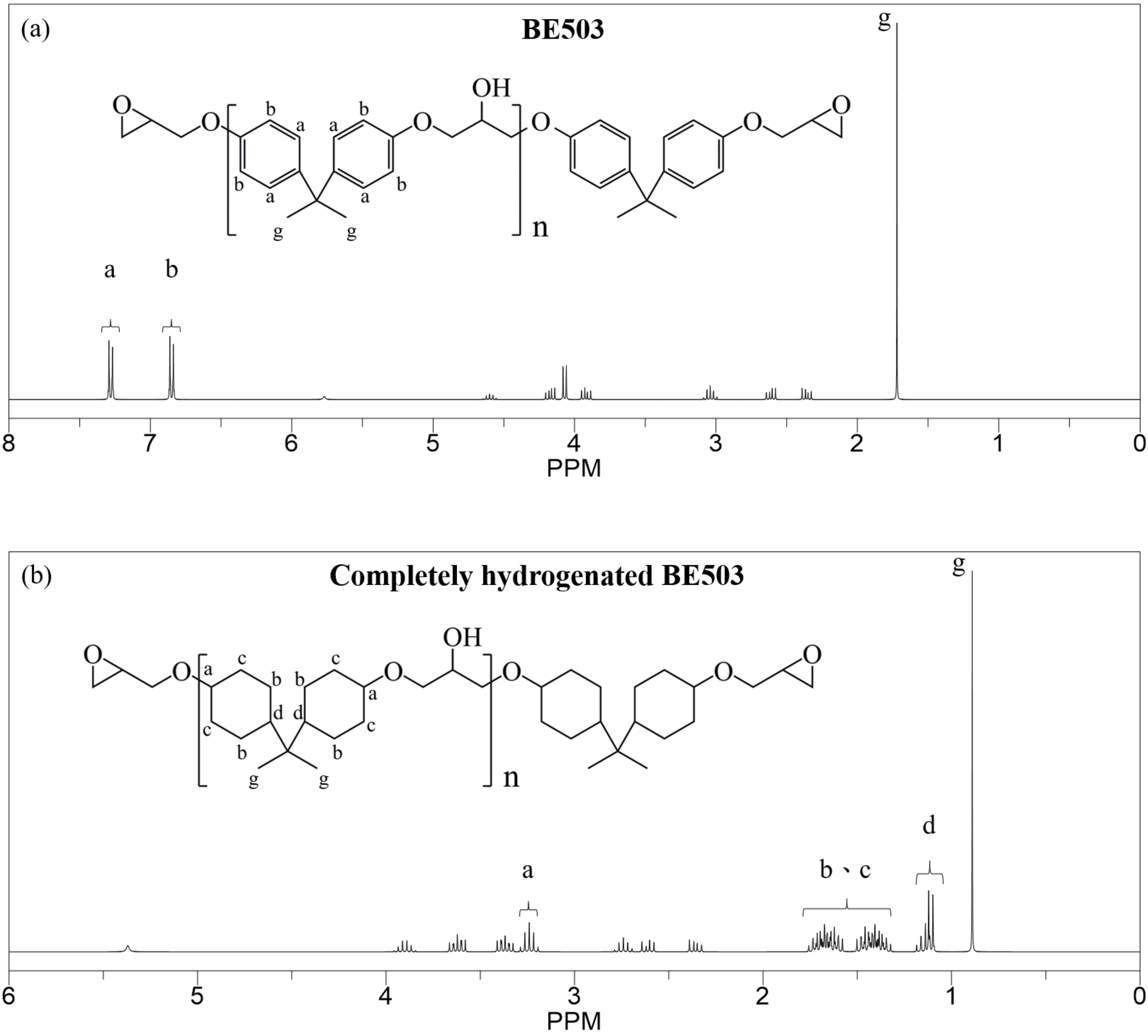
(a)
BE503
OH
n
a
b
g
8
7
6
5
4
3
2
1
0
PPM
(b)
Completely hydrogenated BE503
OH
n
a
b、c
d
g
6
5
4
3
2
1
0
PPM

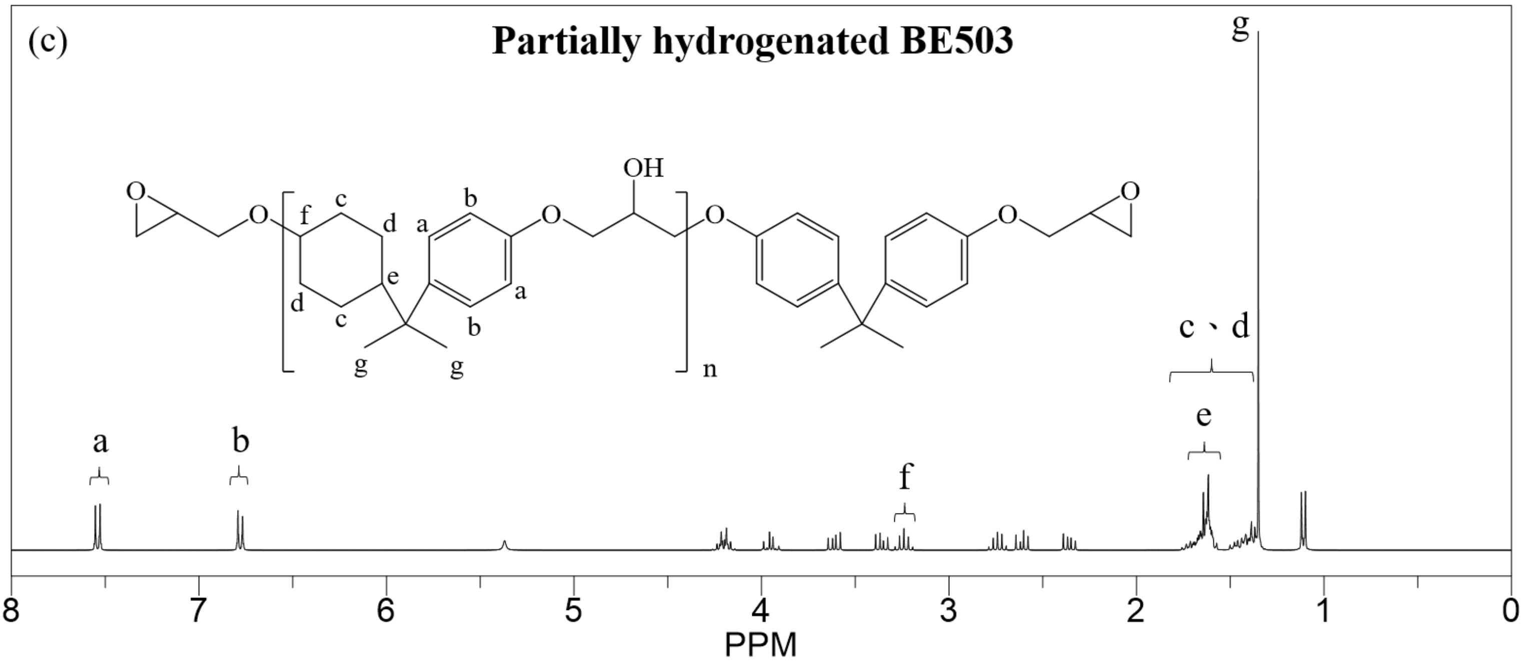

From Figure S2c as a typical example:

$$hydrogenation\ yield\ (\%) = \frac{\frac{c+d+e}{9}}{\frac{c+d+e}{9} + \frac{a+b}{4}} \times 100$$

Where:

a, **b** is peak area of H_2 on unsaturated aromatic ring

c, **d**, **e** is peak area of H_2 on saturated aromatic ring

9 = total number of H_2 on saturated aromatic ring except from peak **f**

4 = total number of H_2 on unsaturated aromatic ring

(the peak **f** is neglected as it is hard to be isolated from other peaks)

(g is attributed to the methyls of the tert-butyl group that did not take part in reaction and was therefore not included in the calculation of hydrogenation yield)

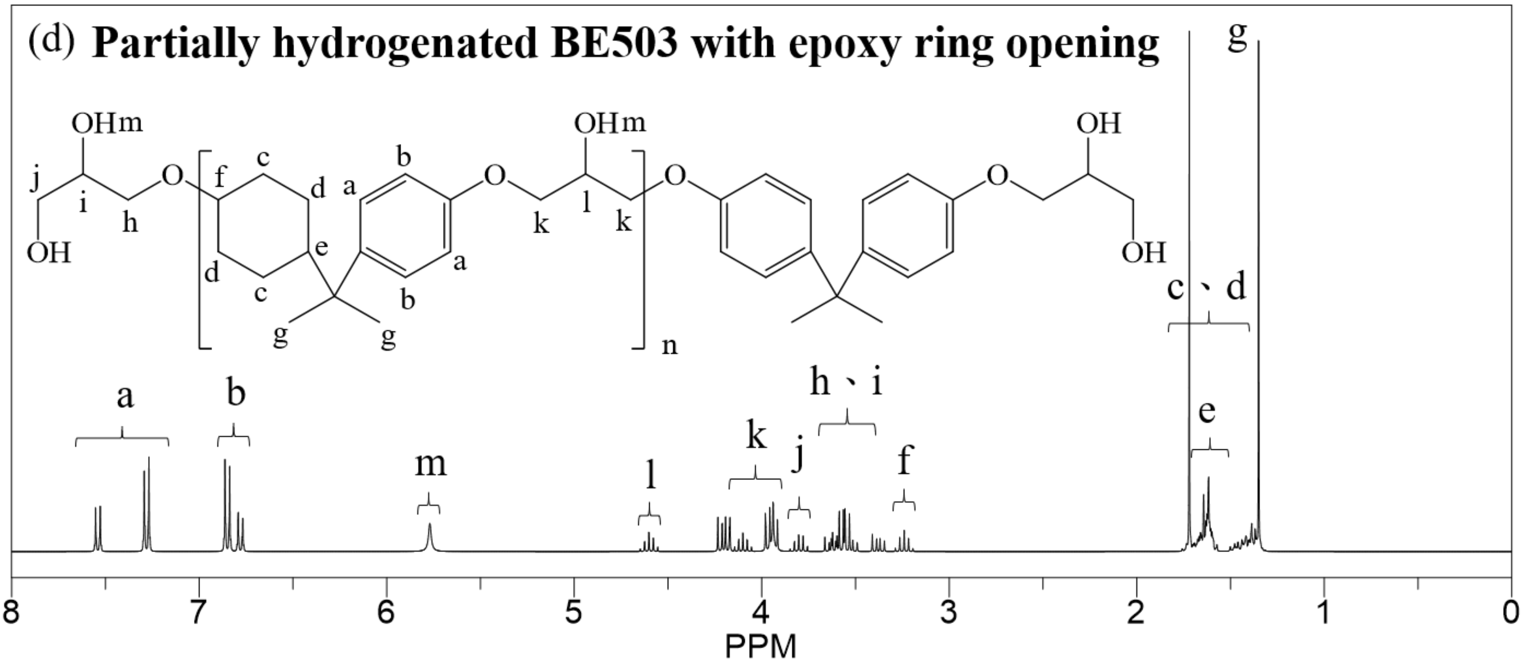

Figure S2. Typical estimations of the ^{1}H NMR spectra of (a) BE503, (b) completely hydrogenated BE503, (c) partially hydrogenated BE503 with calculation for hydrogenation yield and (d) partially hydrogenated BE503 with epoxy ring opening.

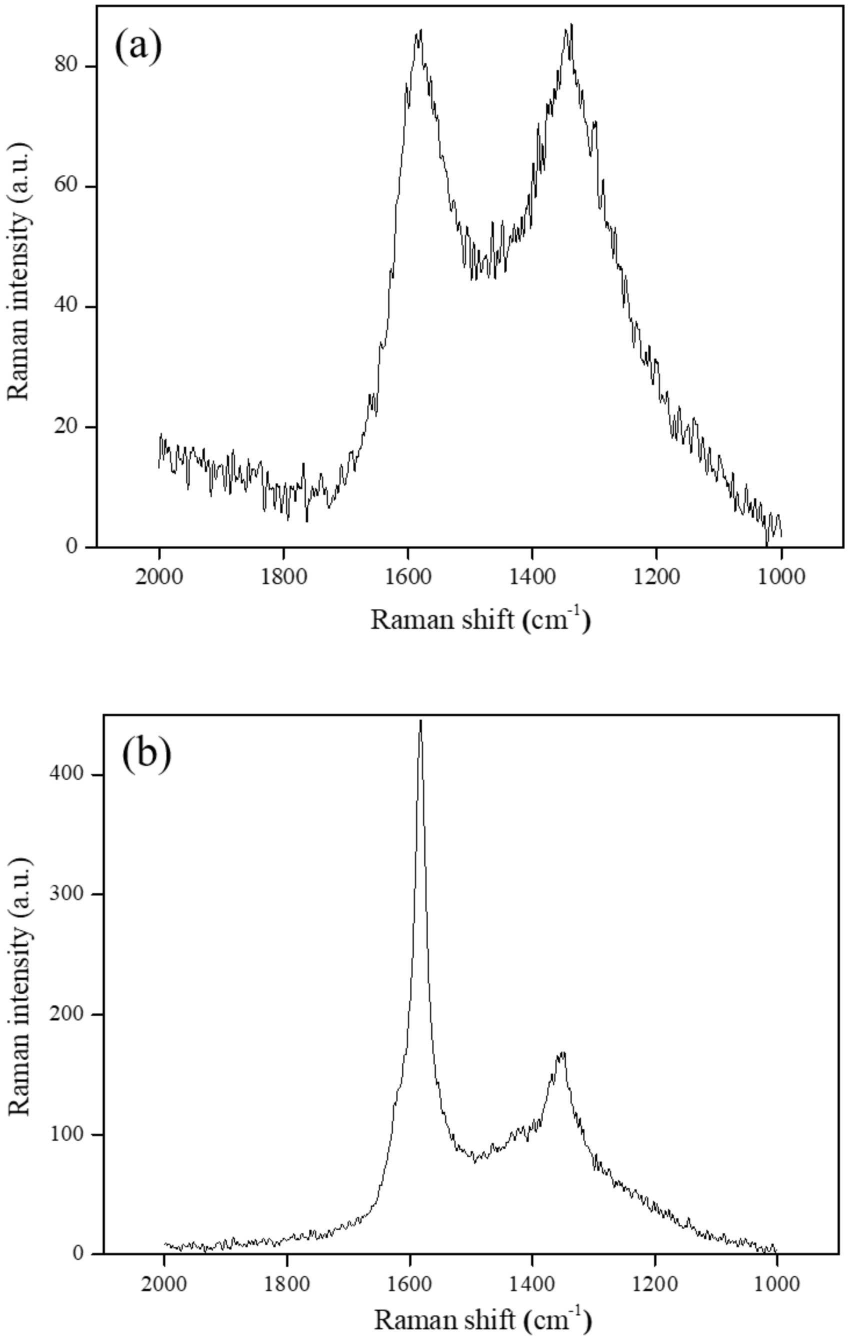
(a)
80
60
40
20
0
Raman intensity (a.u.)
2000
1800
1600
1400
1200
1000
Raman shift (cm-1)
(b)
400
300
200
100
0
Raman intensity (a.u.)
2000
1800
1600
1400
1200
1000
Raman shift (cm-1)

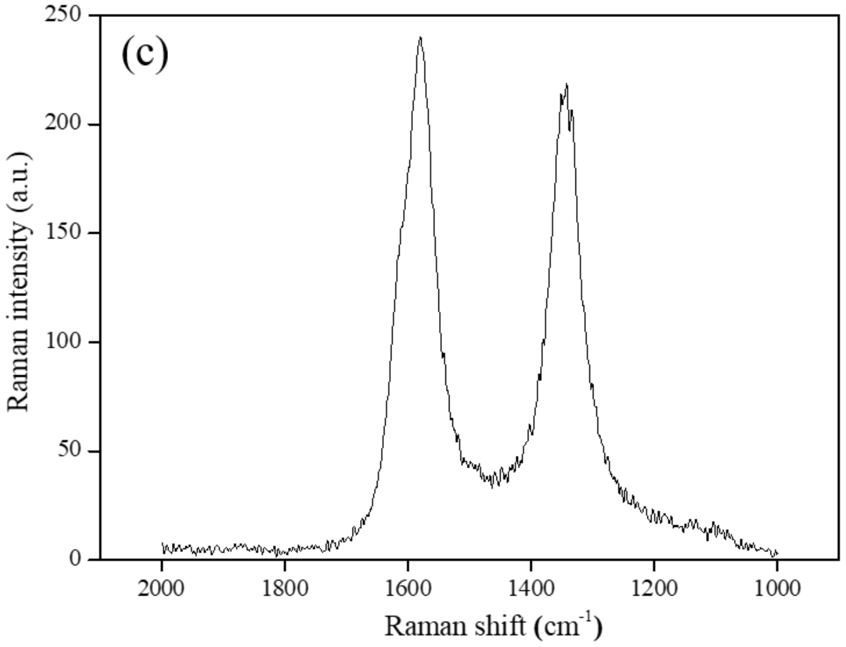

Figure S3. Raman spectra of: (a) VulcanXC72, (b) Graphene and (c) MWCNTs.

References

1. Kamlet, M.J.; Abboud, J.L.M.; Abraham, M.H.; Taft, R.W. Linear solvation energy relationships. 23. A comprehensive collection of the solvatochromic parameters, pi.*, .alpha., and .beta., and some methods for simplifying the generalized solvatochromic equation. *The J. of Org. Chem.* **1983**, *48*, 2877-2887.
2. Zrnčević, S.J.C. Kinetics and mass transfer in the hydrogenation of 2-((1-benzyl-1,2,3,6-tetrahydropyridin-4-yl)methylene)-5,6-dimethoxy-2,3-dihydroinden-1-one hydrochloride over Pt/C Catalyst. *Chem. Biochem. Eng. Q.* **2015**, *28*, 437-445.

© 2020 by the authors. Submitted for possible open access publication under the terms and conditions of the Creative Commons Attribution (CC BY) license (http://creativecommons.org/licenses/by/4.0/).
